# Notes and News

**Published:** 1900-07-28

**Authors:** 


### NOTES AND NEWS.

A new development in the meat trade is promised us, it having been discovered that it is possible to preserve meat by keeping it in sterilised air, or in a special chamber. If this means that the air is sterilised by heat or by filtration, or some such innocuous process, this is good news, for it would be proof that the meat was clean and in good condition when it entered the chamber. If, on the other hand, the "sterilised air" means air rendered poisonous to flies, microbes, and other destructive creatures by aid of chemicals, then we do not feel drawn to it.

The Governor of Hong Kong telegraphed last Monday to the Colonial Office saying that 44 deaths from plague had taken place last week, and that 43 cases had been reported. Plague still prevails in Senegal, there having been seven deaths at Dakar and Gorée last Saturday; and a suspicious death occurred on board the "Ville de Pernambuco," which arrived at Panillac on Monday with 50 passengers from Dakar. The Colonial Office has also received an official report from Sydney stating that on July 14th 51 cases remained under treatment, the total cases having numbered 300.

On Tuesday a deputation from the Royal College of Surgeons of England attended at Marlborough House to present the Diploma of Honorary Fellowship of the College to H.R.H. the Prince of Wales. The three following Honorary Fellows have already been elected: H.R.H. the Prince of Wales, K.G., who was elected on June 14th, and the most Hon. the Marquis of Salisbury, K.G., and the Right Hon. the Earl of Rosebery, K.G., K.T., who were elected on July 12th. Quite a string of notable surgeons, including many foreigners, are also being elected to the Honorary Fellowship in celebration of the Centenary Festival of the Royal College.

The Royal College of Surgeons celebrates its centenary this week by a series of ceremonies and festivities, the principal of which are the centenary meeting in the theatre of the University of London, Burlington Gardens, at which the Prince of Wales will be present, when the various honorary Fellows will receive their diplomas; the dinner on Thursday evening in the hall of the Honourable Society of Lincoln's Inn; and the conversaziones on that and the following evening.

Up to the end of last week the total receipts of the Hospital Sunday Fund amounted to nearly £48,000.

Sir William MacCormac has been unanimously elected President of the Royal College of Surgeons for the fifth time in succession.

A reception was given last Monday to the members attending the thirty-seventh annual meeting of the British Pharmaceutical Conference.

The very high temperature which is prevalent in the United States is making people there very anxious with regard to the presence of yellow fever in the coast towns of Central and South America. So far as the States are concerned Vera Cruz is the centre of infection most to be dreaded.

Hot days we can stand, but when the nights are hot as well the trial is great indeed. Last Sunday night the highest minimum night temperature occurred that has been recorded during the 43 years that observations have been taken at 62, Camden Square. During the whole of that (Sunday to Monday) night the temperature only once dropped to 67·7 deg. Fah. on the Glaisher stand, and 68·1 deg. Fah. in the Stevenson screen.

In view of the number and the gravity of the accidents which arise from boiler explosions, interest attaches to the report of the Select Committee of the House of Commons appointed to inquire into the advisability of legislation to ensure the systematic inspection of boilers. In their report the Committee state that, although they are satisfied that explosions occur more frequently in uninspected than in inspected boilers, the evidence goes to show that explosions also often occur from mistakes or incapacity on the part of attendants which inspection would not prevent. They do not then recommend the appointment of inspectors under the Board of Trade, holding that the owner should not have the opportunity of sheltering himself from responsibility behind an official inspection. They prefer an extension of the Factory Acts, empowering an inspector to call upon the user of a boiler to produce a certificate, showing that he has had his boiler inspected by a competent person within a year.

Some of the saner people in America are beginning to recognise the madness of their fellow-citizens, the special madness that just now attracts their attention being the method which the Americans adopt of demonstrating their patriotism on the national fête day. According to reports received from 125 cities, says the New York *Medical Record*, 30 persons were killed, and 1,325 were injured this time. Out of this total 442 can attribute their injuries to firecrackers and dynamite torpedoes. The deadly cannon firecracker did most of this execution. The New York *Medical News*, commenting on the same subject, says that seven millions of dollars went up in the blaze and smoke and noise of fireworks. "Fingers, hands, eyes, and lives were freely sacrificed. In Philadelphia seven children in one group lost their lives while indulging in these Oriental orgies. At one rest-side dispensary in New York 350 cases of burns and gunshot wounds were dressed on July 5th." Surely all this is unworthy of an educated and civilised condition. Not that we dare to criticise. After Mafeking night we are dumb. If we had had the crackers handy there is no knowing what we might not have done.

